# Flexor tenodesis procedure in the treatment of lesser toe deformities

**DOI:** 10.1007/s00402-021-03942-9

**Published:** 2021-05-11

**Authors:** Cesar de Cesar Netto, Eli L Schmidt, Matthieu Lalevee, Nacime Salomao Barbachan Mansur

**Affiliations:** 1 Department of Orthopaedic and Rehabilitation,Carver College of Medicine, University of Iowa, 200 Hawkins Dr, John Pappan John Pavillion (JPP), Room 01066, Lower Level, Iowa City, IA 52242 USA; 2 Department of Orthopedics and Traumatology, Paulista School of Medicine, Federal University of Sao Paulo, Sao Paulo, SP Brazil

**Keywords:** Hammertoe, Hammertoe deformity, Toe deformity, Tenodesis, Tendon transfer

## Abstract

**Abstract:**

In this technical report study, we describe the use of a flexor tenodesis procedure in the treatment of lesser toe deformities (LTD). Using a specific implant, both the flexor digitorum longus and brevis tendons are attached to the plantar aspect of the proximal phalanx, allowing dynamic correction of flexible deformities of metatarsophalangeal and interphalangeal joints. Good clinical results and absence of complications were observed in a series of 3 patients, with considerable correction of the LTD, and absence of substantial residual floating toe or metatarsophalangeal joint stiffness.

**Level of evidence:**

V – Technical Report/Case Report/Expert Opinion.

## Introduction

Lesser toe deformities (LTD) and metatarsalgia are prevalent and challenging conditions that affect up to 60% of the adult population [1–4]. Associated pain and footwear-related problems frequently lead symptomatic patients to seek medical attention [5]. Many non-surgical options such as footwear modification, taping and bracing have been proposed. However, operative treatment is frequently needed particularly in the rigid deformities due to the progressive nature of the problem, footwear restrictions, development of callosities/sores/ulcerations in prominent areas of the toes, and incapacitating symptoms [6–10].

Several surgical techniques and surgical treatment algorithms have been proposed in the literature to address LTD, usually based on the type (hammer toe, claw toe, mallet toe), severity and stiffness of the deformities [9, 11–13]. Techniques can be broadly divided into soft-tissue release/rebalancing, osteotomies and arthrodesis, or a combination of them [9]. Most of the techniques carry a considerable rate of complications with only fair to good clinical results, and there are some risk factors for worse outcomes reported in the literature [14–16]. Distal lesser metatarsal shortening osteotomies (DMSO) are probably the most frequently performed procedure when the metatarsophalangeal joint (MTPJ) is involved in the deformity, usually with increased dorsiflexion. The Weil osteotomy is perhaps the standard DMSO technique and consists of an intra-articular oblique osteotomy aligned parallel to the floor [17]. The reported complications of DMSO include recurrence of the sagittal plane component of the deformity, with floating toes presenting in up to 68% of the cases [18, 19], as well as frequent and considerable MTPJ stiffness. The intraarticular or intracapsular characteristic of these osteotomies, associated with the need for extensive dorsal soft tissue dissection, is deemed to be partially responsible for the prevalence of these complications [19]. Modifications of the technique such as the Maceira modification, an extra-articular MSO, and the percutaneous DMSO, popularized as the distal mini-invasive metatarsal osteotomy (DMMO), have also been proposed aiming to mitigate associated complications reported with the index procedure [20, 21].

Soft tissue procedures such as capsular and plantar plate repair/imbrication as well as tendon balancing techniques have also been utilized with the aim of minimizing some of these complications [22–26]. Flexor to extensor tendon transfers are frequently used soft tissue techniques that take the advantage of using a flexor digitorum longus (FDL) tenodesis into the base of the proximal phalanx, benefiting from the resultant dynamic effect of pulling the proximal phalanx plantarly, maintaining the toe in contact with the floor and avoiding the FDL deforming force on the distal phalanx [27, 28]. Even though the reported clinical outcomes of this procedure for the treatment of flexible LTD are good, reported complications are similar to the other techniques, with the stiffness of the toe and MTPJ being frequent [29, 30]. These complications are possibly explained by the relatively extensive plantar and dorsal dissection needed, as well as by the frequently performed temporary adjunctive K-wire fixation of the toe and MTPJ [31–33]. The use of provisional K-wire fixation to maintain the straight toe and the MTPJ in relative plantarflexion during the healing process, usually for 3–6 weeks, has been associated with morbidity and several complications including toe vascular insufficiency, wire loosening/bending/breakage, impaired toe perfusion, or pin tract infection [31–33]. New minimally invasive techniques have been gaining popularity, with the objective of minimizing surgical dissection and avoiding the need for K-wire fixation [34].

The aim of this study is to describe an alternative surgical technique of a minimally invasive flexor tenodesis procedure to correct LTD, particularly its MTPJ component, and fixation with a relatively new implant, as well as to report our early experience and clinical results in a small cohort of patients with LTD that were treated with this technique.

## Methods

### Design and Sample

This is an IRB-approved technical-tip and retrospective case report study. All patients that underwent flexor tenodesis as part of surgical treatment of LTD from January 2020 to December 2020 using the specific surgical technique and implant were included (three patients, three feet, 2 left and 1 right). All patients had failed at least 6 months of non-operative treatment. Procedures were performed by a single fellowship-trained orthopedic foot and ankle surgeon with over 10 years of experience.

### Surgical Technique Flexor Tenodesis

**Fig. 1:**
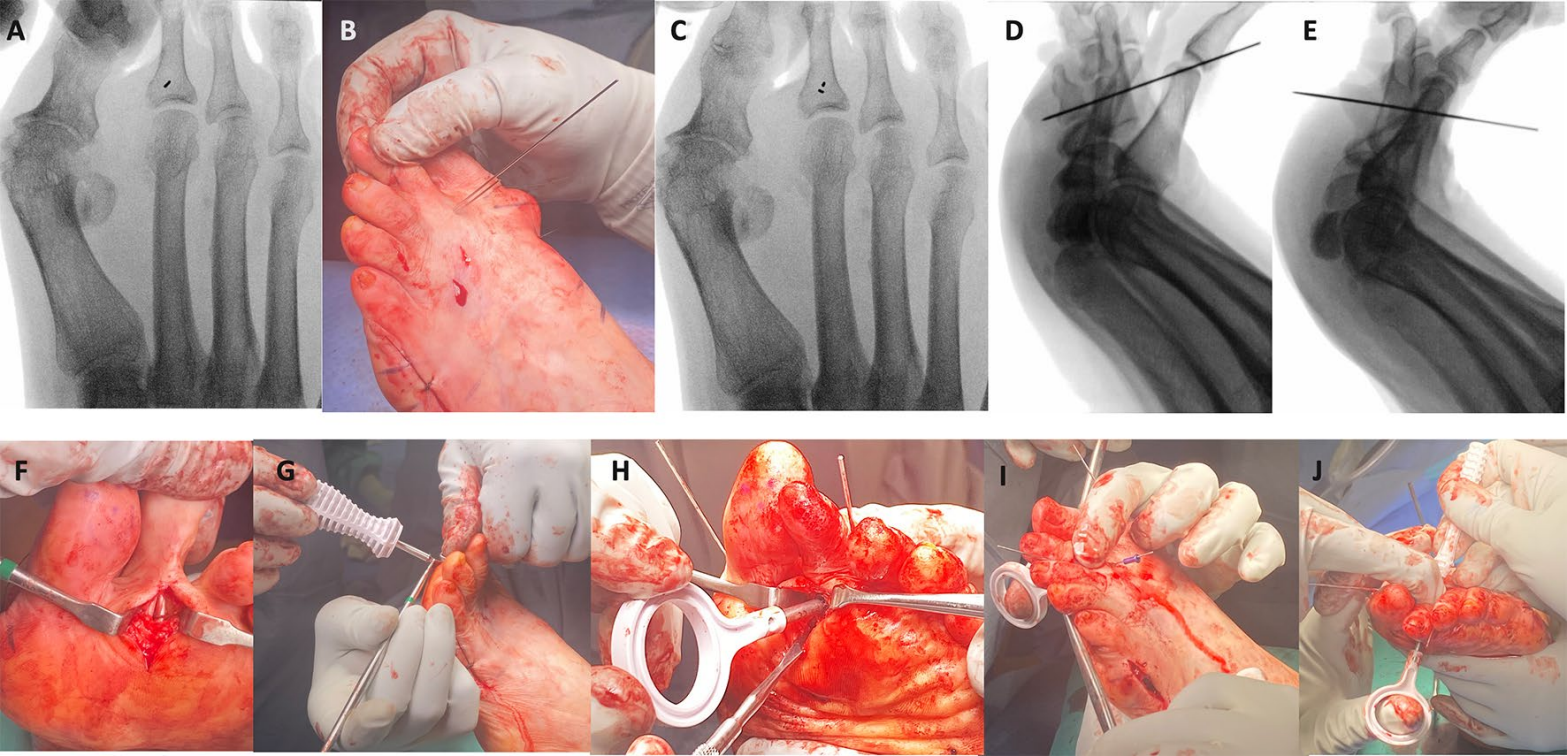
Flexor Tenodesis Surgical Technique for the 2nd Toe. **a** Under fluoroscopic guidance, a K-wire is used to find the level of the base of the proximal phalanx so the dorsal small direct approach can be performed. Ideally, the K-wire should be positioned in the central aspect of metaphyseal area of the proximal phalanx. **b**-**c** A second K-wire can be inserted also under fluoroscopic guidance, using the first one as a landmark for ideal positioning. **d** On the lateral fluoroscopic view, the ideal positioning is so the K-wire should be perpendicular to the plantar aspect of the proximal phalanx. **e** Since during the first attempt the K-wire was aimed to proximally, it was repositioned and is now more perpendicular to the plantar surface of the proximal phalanx. **f** Once ideal K-wire positioning is achieved on anteroposterior and lateral fluoroscopic views, the wire is advanced plantarly through the skin, and a plantar longitudinal approach is developed to expose the K-wire, opening the flexor tendon sheath and making sure the wire is positioned in between the two slips of the flexor digitorum longus tendon (FDL). **g** The manual reamer/drill bit is inserted over the guidewire from plantar to dorsal first, while an assistant retracts the flexor tendons. **h** The male implant is inserted from plantar to dorsal using the appropriate introducer. Under direct visualization, the spikes of the implant should be grasping both slips of the FDL and Flexor Digitorum Brevis Tendon. **i** The female component of the implant is inserted from dorsal to plantar through the tip of the introducer. **j** While keeping the interphalangeal joints in full extension, and the metatarsophalangeal joint in plantarflexion, the female component of the implant is tightened dorsally into the male implant

The patients underwent general anesthesia and a peripheral popliteal block procedure. They were positioned supine on the operating table. Appropriate preparation and draping were performed. A 5 mm midline dorsal longitudinal approach was performed at the level of the proximal third of the proximal phalanx of the second toe. Blunt dissection was caried down to bone, retracting the extensor tendons laterally/medially. Under fluoroscopic guidance, a 0.9 mm guidewire was introduced from dorsal to plantar at the central aspect of the proximal metaphyseal area of the proximal phalanx of the second toe. A second guidewire can be utilized using the first one as a reference if the surgeon is not happy with the positioning (Fig. 1a, b and c). The sagittal plane positioning of the guidewire is such that the wire is perpendicular to the plantar surface of the proximal phalanx (Fig. 1d and e). A plantar longitudinal incision of 3 cm is then performed at the plantar exit point of the guidewire. Blunt dissection is performed through the subcutaneous tissue down to the level of the flexor tendons sheath. The sheath is sharply incised longitudinally exposing the flexor tendons. The guidewire is retracted dorsally and then pushed forward again under direct visualization of the flexor tendons, making sure that the trajectory of the wire is in between the two slips of the flexor digitorum longus tendon (Fig. 1f).

A manual 2.8 mm reamer is then used from plantar to dorsal over the guidewire to create a tunnel in the proximal phalanx while protecting the flexor tendons (Fig. 1g). The same reamer is then utilized to ream the same tunnel from dorsal to plantar, removing some possible extra bone on the dorsal aspect of the phalanx. Manual correction of the toe deformity is performed with the help of an assistant. The interphalangeal joints are supposed to be positioned in maximal dorsiflexion aiming to provide tensioning of the FDL and FDB tendons of the second toe, maximizing the dynamic effect of the correction. A 0.045 K-wire can be provisionally introduced from distal to proximal into the second toe, crossing the IP joints, maintaining the reduction and facilitating the introduction of the implant. This provisional fixation can be removed at the end of the procedure. The MTPJ should be manually reduced in the desired position, usually with slight plantarflexion. From the plantar approach, and under direct visualization of both slips of the FDL and FDB, a male track implant that contains a row of spikes to grab the tendons and push them towards the plantar aspect of the proximal phalanx is then inserted using the inserter for the male tack (Fig. 1h), grasping both slips of the FDL and the FDB. While keeping the reduction, the size of the female sleeve implant is measured from the dorsal approach and then the appropriately sized female implant is inserted dorsally and screwed into the male implant using the proper screwdriver (Fig. 1i and j), compressing the tendons and the spikes of the male implant into the plantar aspect of the base of the proximal phalanx.

Before inserting the female implant, additional axial tensioning of the tendons pulling them distally can be performed by an assistant using a hemostat to optimize correction. The medial and lateral slips of the FDL can also be pulled asymmetrically for correction of eventual axial plane deformities of the toe, for example applying more tension into the lateral slip of the tendons to correct a medial deviation of the lesser toe, and the medial slip for correction of a lateral deviation.

**Fig. 2:**
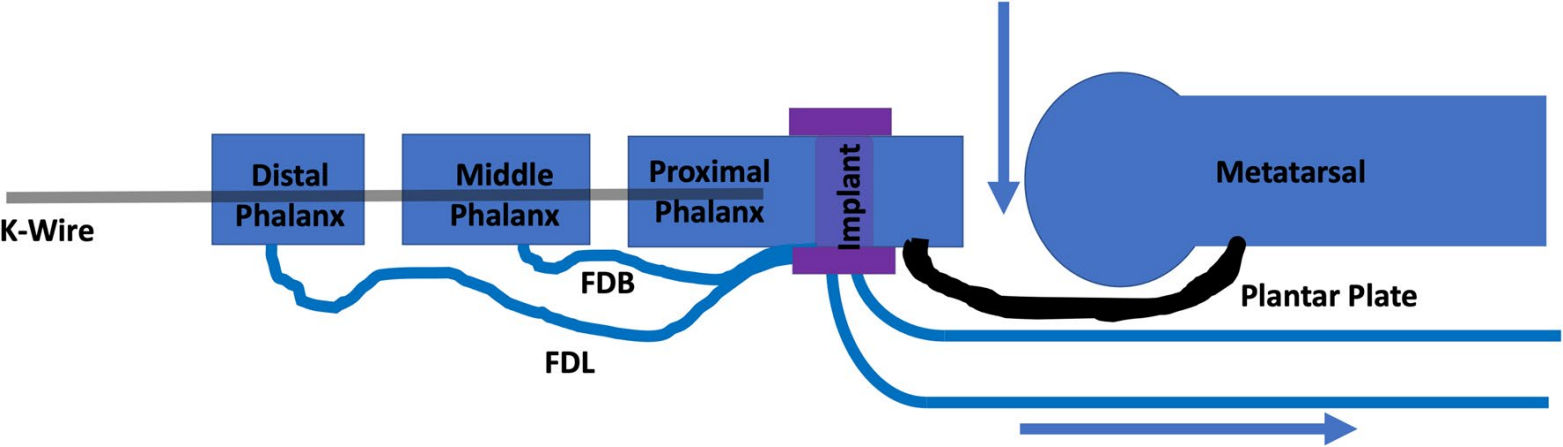
Representative drawing of flexor tendons (Flexor Digitorum Longus, FDL, and Flexor Digitorum Brevis, FDB, Tendons) after flexor tenodesis to the plantar aspect of the proximal phalanx is finalized. With provisional K-wire fixation retaining the interphalangeal joints in extension, tenodesis implant was inserted in the proximal phalanx, maintaining the distal aspects of FDL and FDB relaxed, and the proximal aspect of the same tendons under tension, pulling the proximal phalanx plantarly

**Fig. 3:**
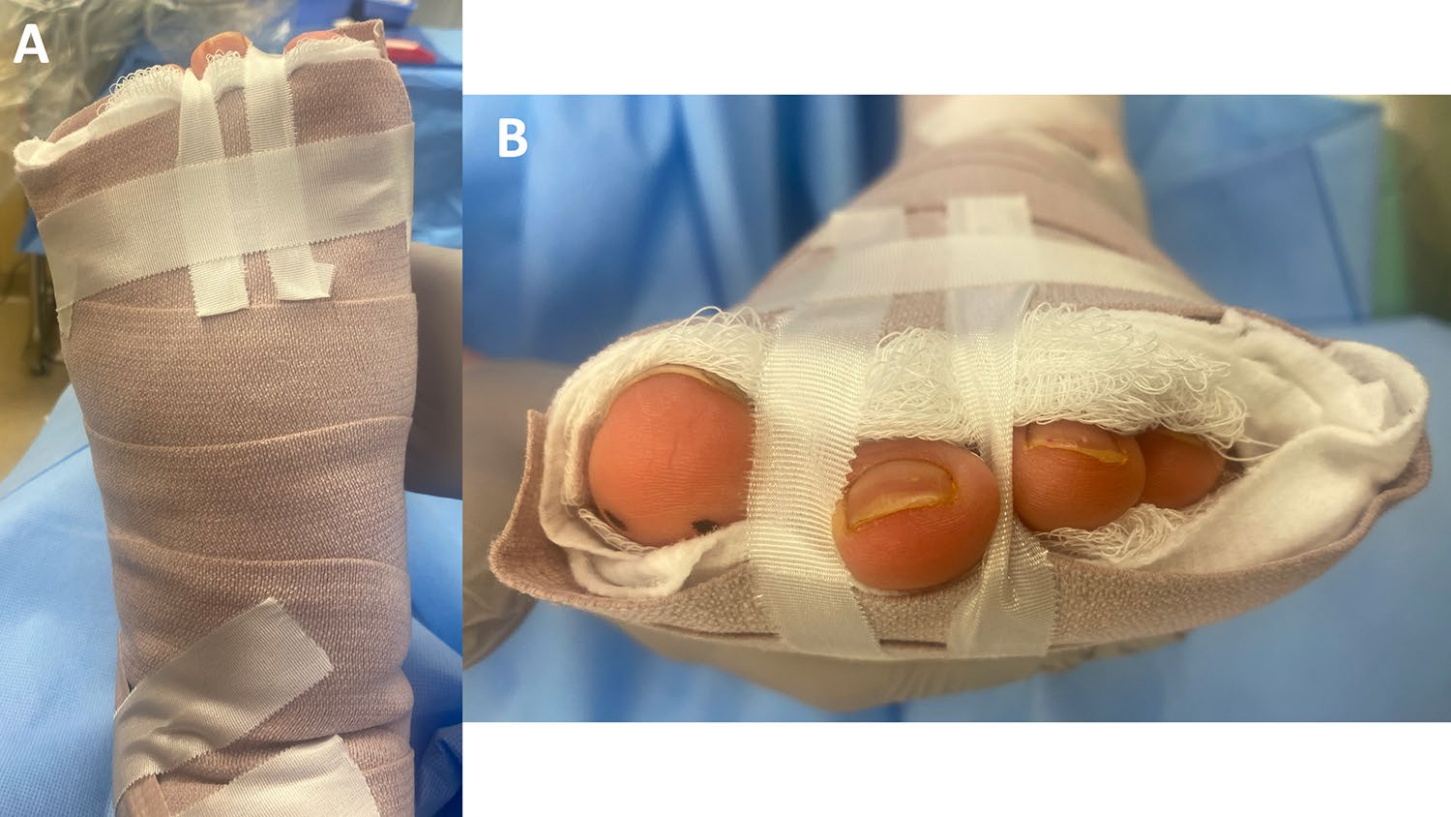
Dorsal (**a**) and distal (**b**) clinical views of an example of compressive “sandwich-like” soft dressing maintaining the relative positioning of the lesser toes, and the strapping of the 2nd toe with plantarflexion of the metatarsophalangeal joint

The amount of correction is checked clinically. If the desired correction was not achieved, the implants can be easily removed and the fixation procedure can be restarted, making sure that the IP joints are in full extension, that both slips of the FDL and FDB are grasped by the implant and that adequate tensioning of the flexor tendons and positioning of the MTPJ are secured. The ideal correction and tensioning of the flexor tendons are depicted in Fig. 2. Once the desired correction is achieved, copious irrigation is performed, and the wounds are closed with preferred sutures. Protection of the correction with a slightly compressive “sandwich-like” soft dressing is paramount, maintaining the correction of the MTPJ deformity by keeping it in plantarflexion (Fig. 3). The patient is placed in a flat post-operative shoe that has a stiffened sole. Heel weight-bearing is allowed during the first two weeks. After 2 weeks if wounds are healed, stitches are removed, and the patient can weight bear as tolerated in the post-operative shoe for an additional 3–4 weeks. After that time, patients can transition into regular shoes. Range of motion (ROM) exercises of the operated toe can be started after 2 weeks, with a passive and active plantarflexion and dorsiflexion of the MTPJ. ROM exercises are imperative for minimizing scar tissue formation, floating toe deformity and stiffness of the MTPJ. Formal physical therapy can be utilized as needed, focusing on neutral to the plantar range of motion and intrinsic muscle strengthening, and can be initiated as early as two weeks postoperatively. As in any other tendon transfer/tenodesis procedure, the dynamic component of the procedure is important for maintaining/improving correction. When patients fire their FDL/FDB tendons, a dynamic plantarflexion pull of the tenodesis at the plantar aspect base of the proximal phalanx will bring the toe down, maintaining correction and reducing the chances of development of dorsiflexion contracture and floating toe deformity.

In cases where additional procedures are required, the osteotomies of the first toe/medial column procedures, lesser metatarsal shortening osteotomies, as well as the eventual PIP fusion/resection arthroplasty procedures should be performed before the final tensioning and fixation of the less toe tenodesis procedure takes place. This is due to the fact that LTD are usually complex deformities and are commonly rigid at the PIP joint or associated with hallux valgus deformity and/or central metatarsalgia, all of which demand surgical correction. Even though here we described the tenodesis procedure for the second toe, the same procedure can likewise be performed to correct deformities of the third and fourth toes.

### Outcomes

Patients were assessed for complications, deformity correction, MTPJ ROM, and function through Patient-Reported Outcome Measurement Information System (PROMIS), Foot Function Index (FFI-R), European Foot and Ankle Society (EFAS) and pain via Pain Catastrophic Scale (PCS).

## Results

Three patients (3 feet: 1 right, 2 left) were operated between January 2020 and January 2021, 2 females and 1 male, with a mean BMI of 29.58 (min 25.22; max 34.58). The mean follow-up was 6.3 months (min 5; max 8).

### Case 1

Fifty-eight-year-old female patient with history of progressive and symptomatic deformity of the second toe. Symptoms were getting worse for the last one year. She had no history of symptoms or deformity in the first toe. She had tried conservative treatment for more than one year with taping, toe sleeves and footwear modification, that were all not enough anymore to control her symptoms. On preoperative physical exam the patient was found to have no hallux valgus deformity, but a moderate first ray instability with a neutral hindfoot alignment. Her LTD consisted of dynamic flexible dorsiflexion deformity of the 2nd MTPJ, and a rigid plantarflexion contracture of the PIPJ. She also had tenderness and thin callosities under the heads of the 2nd and 3rd metatarsals and over the PIPJ of the second toe. Patient underwent DMMO of the second and third metatarsals, PIPJ fusion of the 2nd toe, plantarflexion osteotomy of the medial cuneiform with a 6 mm allograft wedge and tenodesis of the flexor tendons to the plantar aspect of the proximal phalanx of the second toe using the TenoTac™ implant (Paragon 28®, Denver, CO, US).

**Fig. 4:**
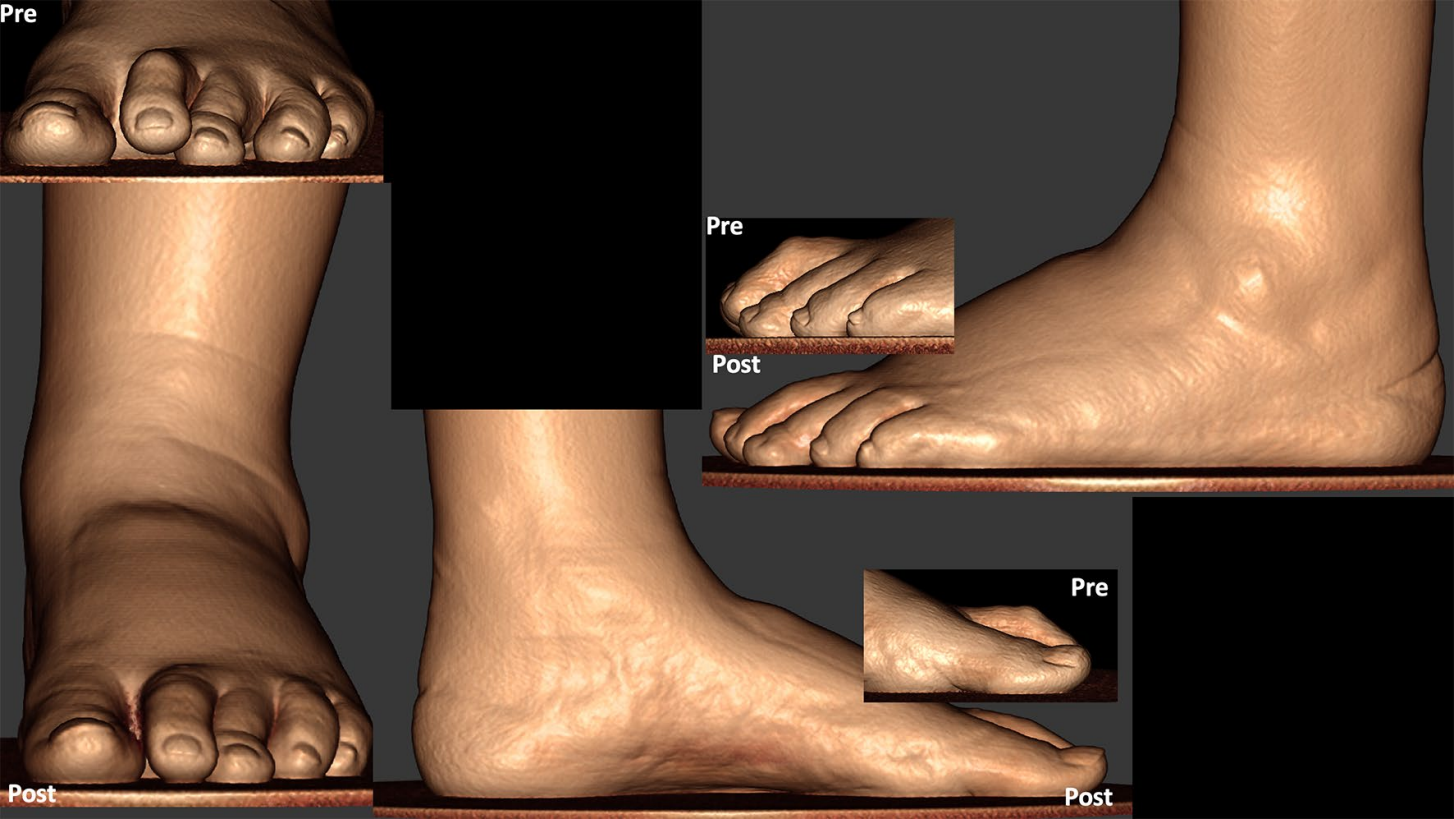
Tree-dimensional surface soft tissue anatomy weight bearing CT reconstruction views of patient’s foot, demonstrating preoperative lesser 2nd toe deformity, and considerable correction 3-months postoperatively

**Fig. 5:**
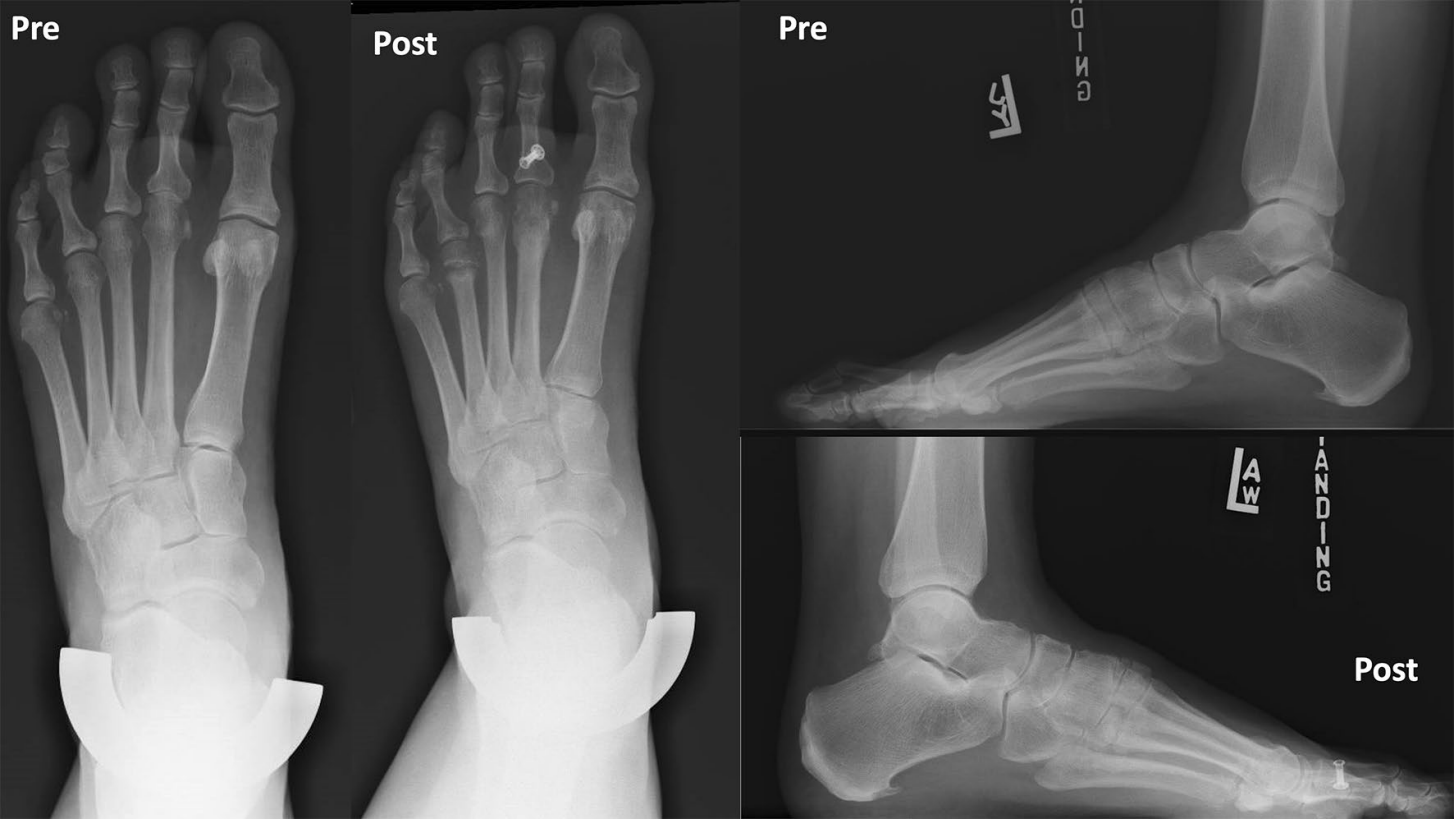
Conventional weight bearing radiographic anteroposterior and lateral views of patient’s foot, demonstrating preoperative lesser 2nd toe deformity with subluxated 2nd metatarsophalangeal joint (MTPJ), and postoperative improved alignment after 6 weeks

**Fig. 6:**
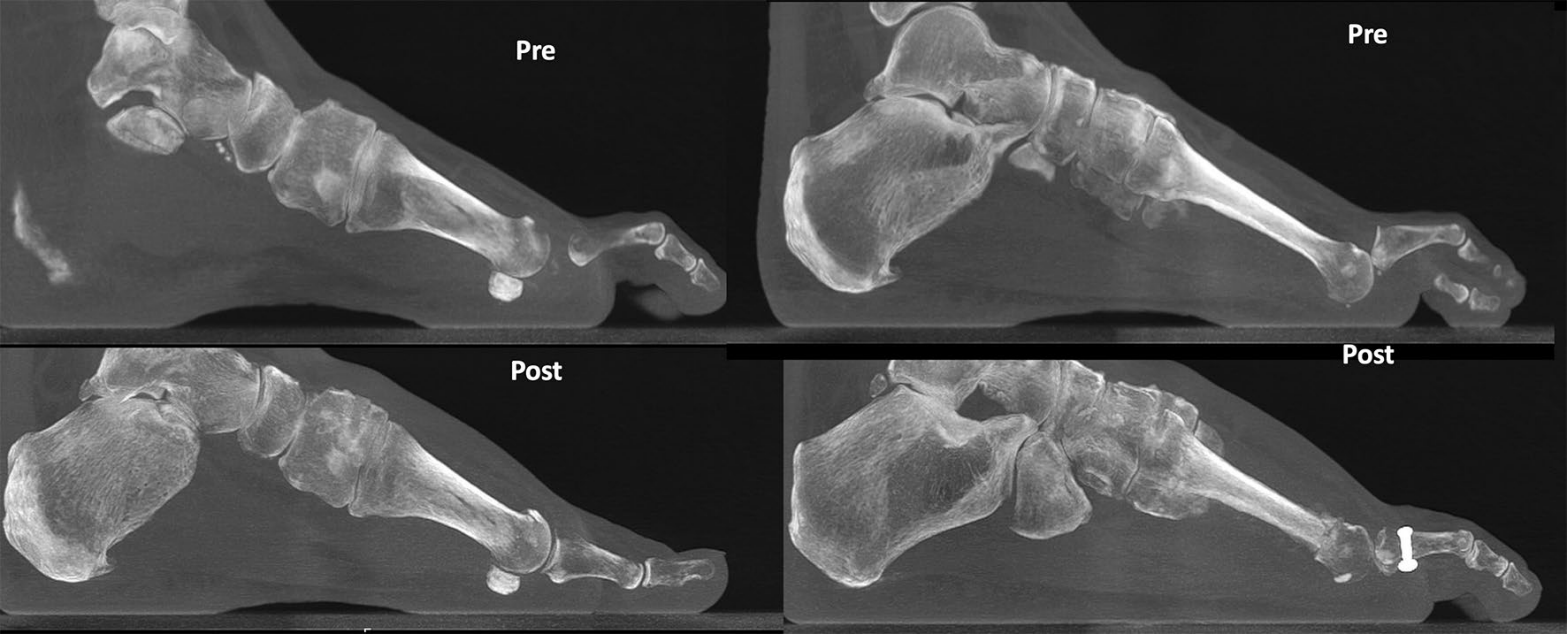
Preoperative and 3-months postoperative wide sagittal weight bearing CT images of patient’s foot 1st and 2nd rays demonstrating preoperative mild collapse of the medial column (Left) as well as lesser 2nd toe deformity and dorsal subluxation of 2nd metatarsophalangeal joint (MTPJ) (Right). Considerable correction of the medial column (Left) and the 2nd toe deformity and MTPJ reduction is postoperatively (Right)

**Fig. 7:**
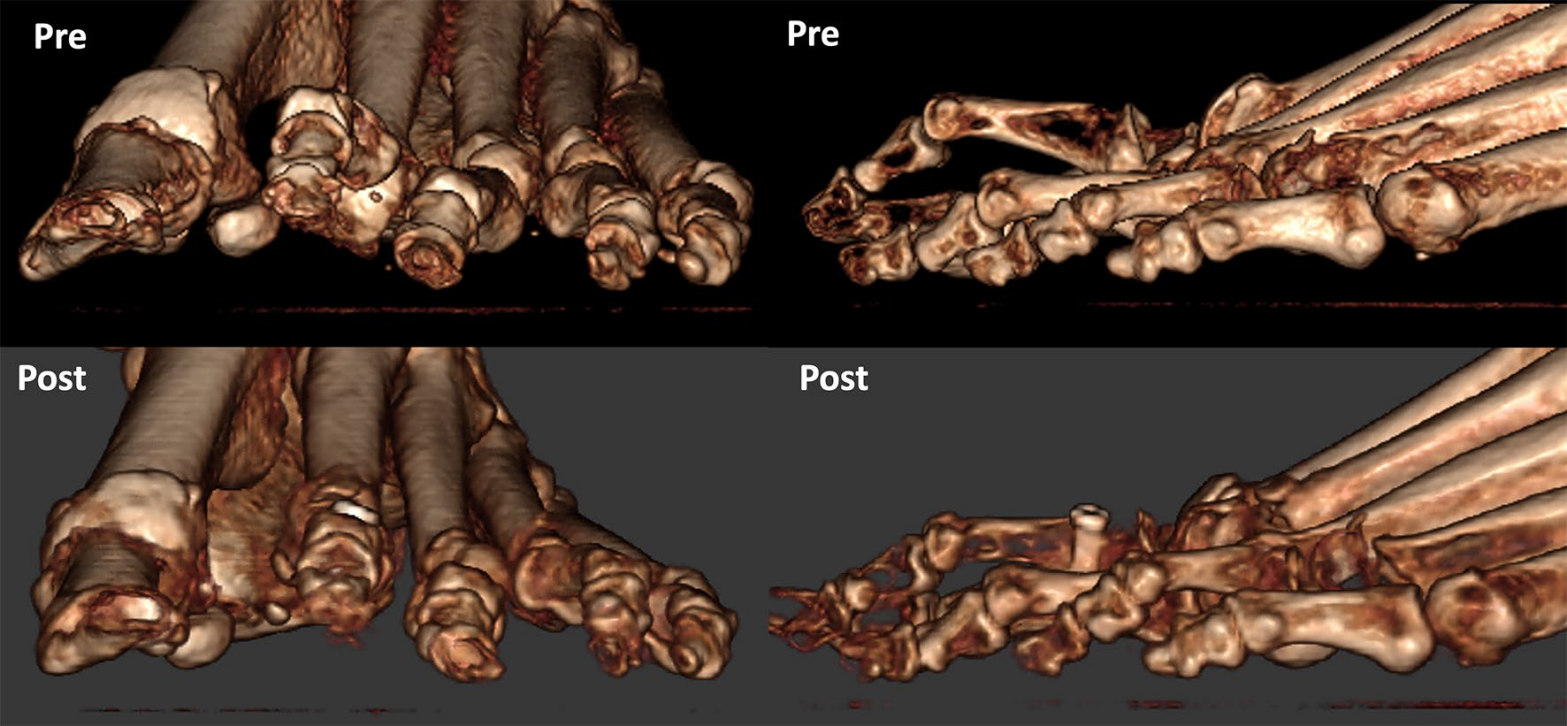
Tree-dimensional surface bone anatomy weight bearing CT reconstruction views of patient’s foot, demonstrating preoperative lesser 2nd toe deformity, and considerable correction 3-months postoperatively

**Fig. 8:**
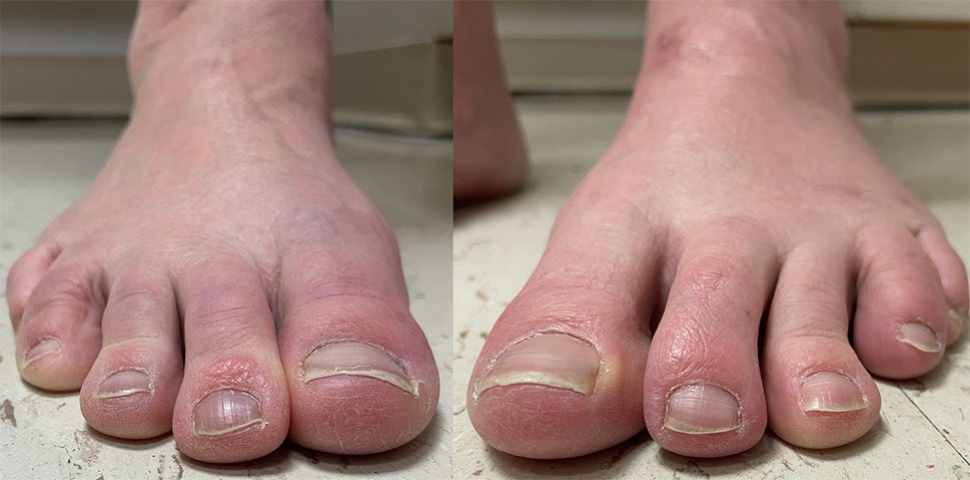
Bilateral forefoot clinical appearance of patient’s toes 6-months postoperatively, demonstrating considerable progressive improvement of the 2nd lesser toe deformity in the left foot, with no signs of the residual floating toe when compared to the right foot

Preoperative and postoperative weight bearing computed tomography (WBCT) images (3-months) and conventional radiographic (6-months) are presented in Figs. 4, 5, 6 and 7. Osteotomies and PIPJ fusion were completely healed after 3 months, with considerable clinical correction of the 2nd toe deformity and resolution of the central metatarsalgia and 2nd toe dorsal symptoms. Clinical correction was even better after 6 months (Fig. 8). Patient ROM of the 2nd MTPJ was almost completely restored, with a total passive dorsiflexion of 60°-70°. She developed no floating toe or dorsiflexion contracture and was extremely satisfied with the procedure. The total clinical follow-up time was 8 months. Patient demonstrated improved clinical outcomes in functional and pain domains, such as PROMIS Physical (29.6 to 44.9), EFAS scale (8 to 13), EFAS Sport Scale (1 to 16), FFI-R (37.5 to 27.2) and PCS (12 to 0).

### Case 2

Seventy-one-year-old male patient with a history of long-term hallux valgus deformity in the right foot. He underwent surgical treatment about 25 years ago, with a distal first metatarsal osteotomy for the HV deformity, with adequate correction for several years. With time, he developed a recurrence of the HV deformity, that got worse progressively, in association with a progressive deformity of the second toe. For about 8 years, he describes a cross-over deformity of the 1st and 2nd toes, with pain symptoms over the medial eminence of the 1st metatarsal head and dorsal aspect of the second toe, as well as some plantar forefoot pain. On physical exam, patient presented with the described cross-over deformity, severe HV 2nd LTD, a rigid ROM of the 1st MTPJ (total ROM of 35–40°), with some pain during ROM. He also had a diffuse callosity over the heads of the 2nd and 3rd metatarsal heads that was painful to palpation, as well as an asymptomatic bunionette deformity. Patient had exhausted conservative treatment options and decided to proceed with surgical treatment. He underwent 1st MTPJ fusion, DMMO of the 2nd, 3rd and 4th metatarsals, and PIPJ fusion with flexor tenodesis of the 2nd toe.

**Fig. 9:**
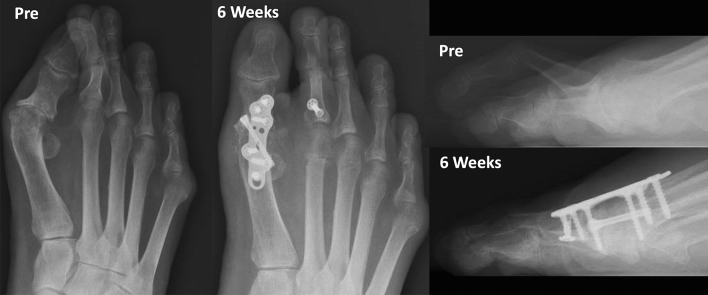
Conventional weight bearing radiographic anteroposterior and lateral views of patient’s foot demonstrating preoperative severe arthritic hallux valgus deformity, and cross-over deformity of the 2nd toe. Postoperative (6-weeks) demonstrating improved alignment of the fused first metatarsophalangeal joint (MTPJ), and considerably improved alignment of the 2nd toe

**Fig. 10:**
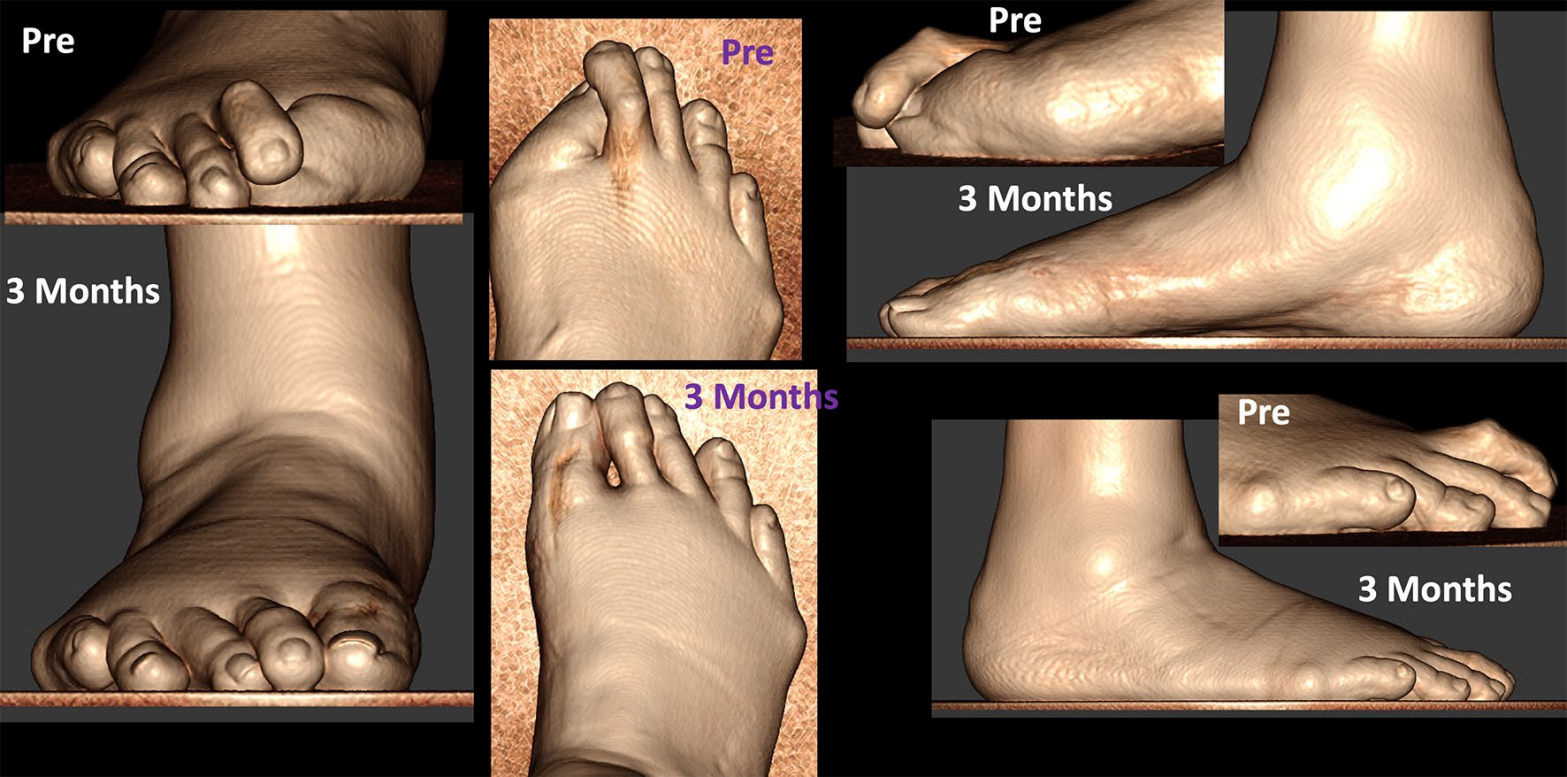
Tree-dimensional surface soft tissue anatomy weight bearing CT reconstruction views of patient’s foot, demonstrating preoperative severe hallux valgus deformity and cross-over lesser 2nd toe deformity, and considerable correction 3-months postoperatively

**Fig. 11:**
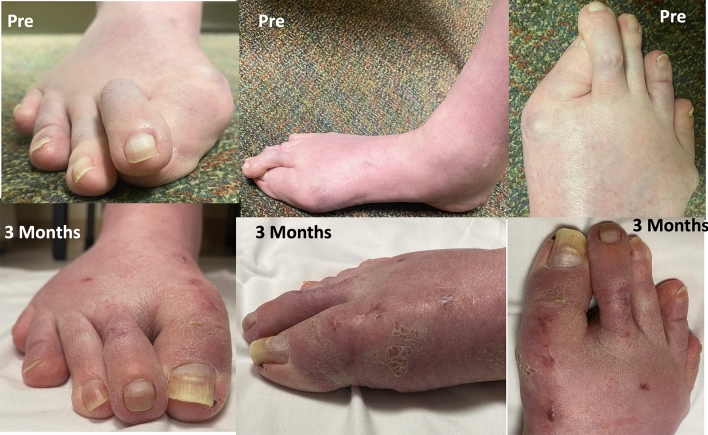
Preoperative and 3-months postoperative clinical pictures of patient’s foot demonstrating preoperative severe hallux valgus deformity and cross-over lesser 2nd toe deformity, and considerable correction postoperatively

Preoperative and postoperative clinical (3 months), weight bearing conventional radiographic (6 weeks) and WBCT images (3 months) are presented in Figs. 9, 10 and 11. WBCT demonstrated completely healed fusions of the 1st MTPJ and PIPJ, as well as the DMMOs. At latest follow-up (6 months), the patient was doing extremely well with a stable 1st MTP joint fusion, well-aligned first toe, and significantly improved sagittal plane alignment of the second toe. He still has residual medial displacement of the lesser toes that was not addressed in the surgical procedure He also is now slightly symptomatic in the bunionette deformity that was asymptomatic preoperatively, which led the patient to decide not to have it corrected at the time of the surgery. The ROM of the second MTPJ is almost completely preserved, with a total of 70o of passive dorsiflexion. There is no residual floating of the 2nd toe. Improvement in clinical outcomes was noted on as PROMIS Physical (42.3 to 47.7), EFAS scale (0 to 3), FFI-R (60.29 to 50) and PCS (4 to 2).

### Case 3

Sixty-three-year-old female patient with a history of at least 10 years of progressive deformity of the first and second left toes. She had never had any surgical treatment. She was able to manage the deformities for a long time with footwear modifications and taping of the second toe, however, symptoms got significantly worse for the last several months. Symptoms are mostly located at the top of the 2nd toe, which has been rubbing on the shoe. On physical exam, the patient was tender on that location, had some mild pain over the medial aspect of the 1st MTPJ, painful callosities underneath the 2nd and 3rd metatarsal heads, a mild-moderate symptomatic bunionette deformity, and a severely unstable first ray. She had exhausted conservative treatment and was looking for surgical treatment options. Among other procedures, she was offered a 1st tarsometatarsal joint fusion to correct the hallux valgus deformity, but refused it, since she did not want any fusions other than a PIPJ fusion to correct her 2nd LTD deformity. She underwent double 1st metatarsal osteotomy to correct the HV, medial proximal opening wedge and distal biplanar chevron-type osteotomy, DMMO of the 2nd, 3rd and 4th metatarsals, PIPJ fusion and flexor tenodesis of the 2nd toe, as well as chevron-type osteotomy of the 5th metatarsal to correct the bunionette deformity.

**Fig. 12:**
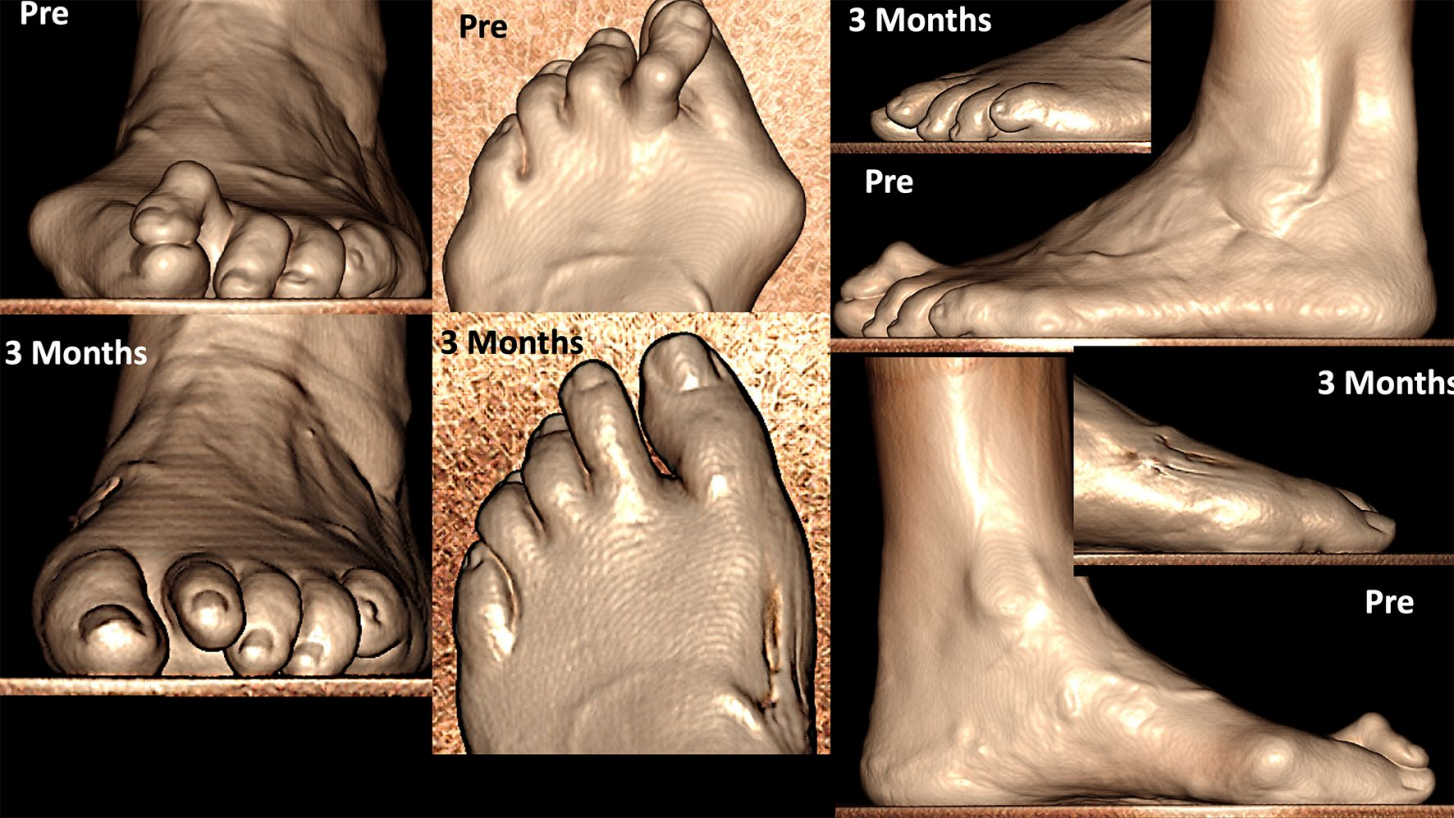
Tree-dimensional surface soft tissue anatomy weight bearing CT reconstruction views of patient’s foot, demonstrating preoperative severe hallux valgus deformity and cross-over lesser 2nd toe deformity, and considerable correction 3-months postoperatively

**Fig. 13:**
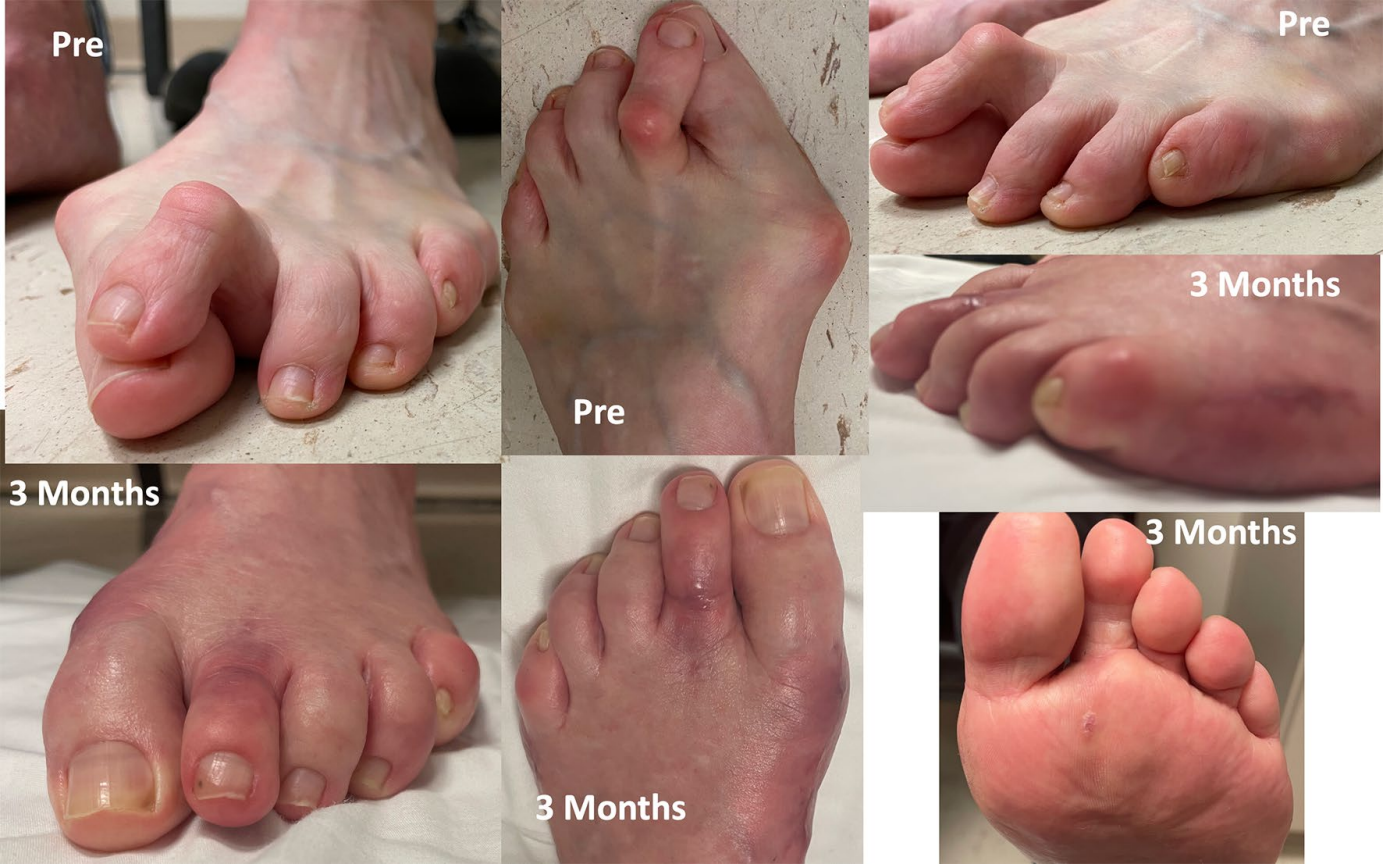
Preoperative and 3-months postoperative clinical pictures of patient’s foot demonstrating severe hallux valgus deformity and cross-over lesser 2nd toe deformity preoperatively, and considerable correction postoperatively, with completely healed plantar and dorsal wounds on the second toe

**Fig. 14:**
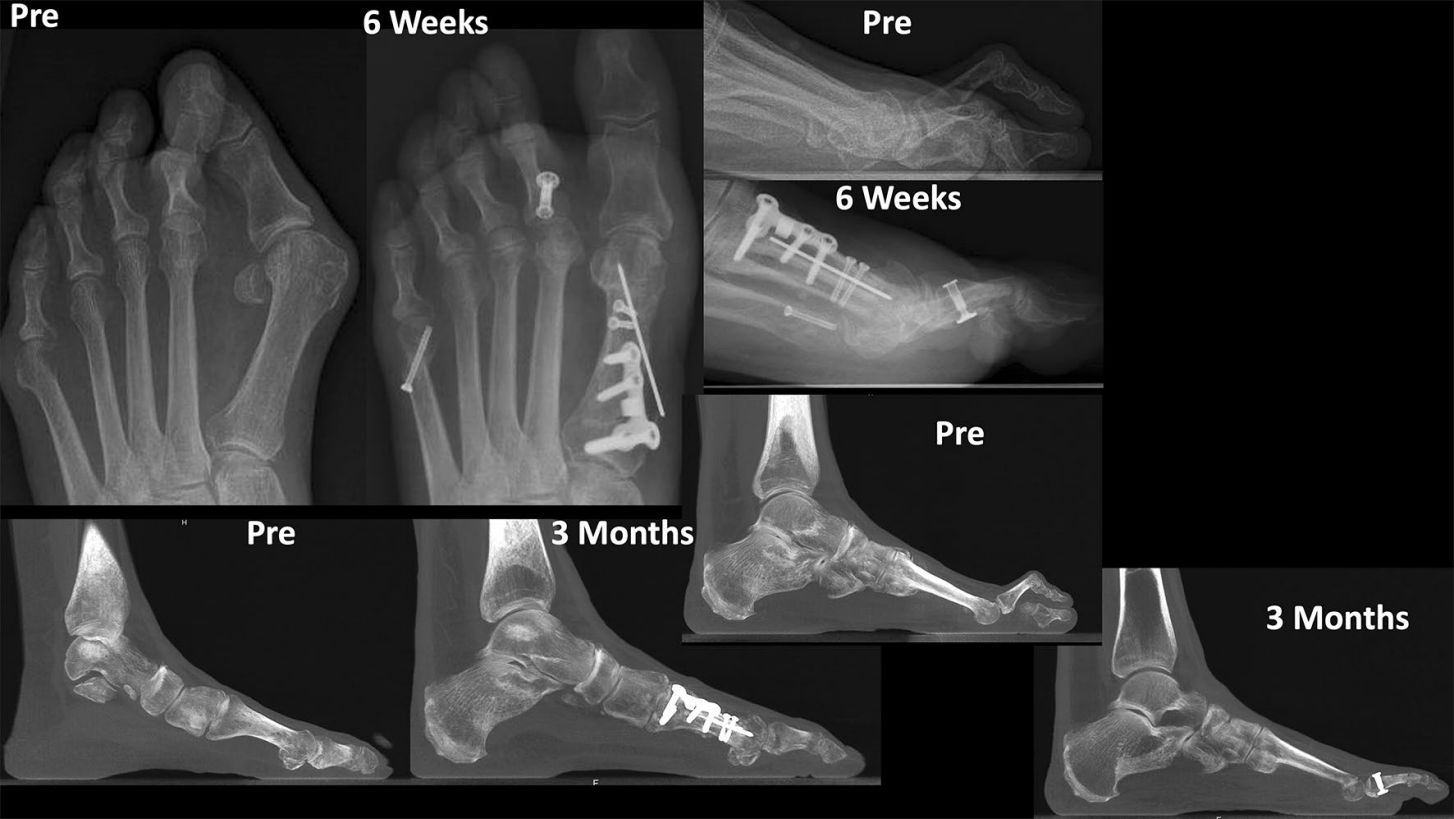
Conventional preoperative and 6-weeks postoperative weight bearing radiographic (anteroposterior and lateral views) of patient’s foot (top), as well as preoperative and 3-months postoperative wide sagittal weight bearing CT images of patient’s foot 1st and 2nd rays (bottom). Demonstration of considerable correction of the hallux valgus and bunionette deformity as well as 2nd toe deformity

Preoperative and postoperative clinical, conventional weight bearing radiographic and WBCT images are presented in Figs. 12, 13 and 14. At 6 weeks, incisions were completely healed, and patient resumed progressive weight bearing. At 3 months, WBCT demonstrated completely healed osteotomies and an adequately healing PIPJ. First, 2nd and 5th toes had considerably improved alignment, with mild stiffness of the 1st and 2nd MTPJ, and a minor residual 2nd floating toe. At the latest follow-up (5 months), the patient was doing better, with improved ROM of the 1st and 2nd MTPJ. The ROM of the second MTPJ is almost completely restored, with a total of 50o of passive dorsiflexion. There is mild residual floating of the 2nd toe, with no pain under the lesser metatarsal heads. Patient demonstrated improved clinical outcomes including PROMIS Physical (27.6 to 42.3), EFAS scale (1 to 4), FFI-R (77.94 to 64.7) and PCS (12 to 6).

## Discussion

Surgical treatment of LTD is challenging and multiple surgical techniques have been described in the literature. Because of the variable reported rates of deformity correction, improvement of symptoms and complications, no technique is accepted as a gold-standard [34, 35]. In this technical-tip study, we described the use of an alternative technique to treat LTD, consisting of a flexor tenodesis procedure of both the FDL and FDB tendons to the plantar aspect of the proximal phalanx of the lesser toes, with a focus on the correction of the sagittal plane dorsiflexion deformity of the MTPJ. We also reported our short-term results utilizing the procedure in a series of three consecutive cases with different severities of 2nd toe deformity. In summary, we observed good clinical and radiographic correction of the LTD with no observed complications related to the surgical technique and implant utilized. Correction was maintained after a minimum clinical follow-up of 5 months and had no important residual floating toe deformity or MTPJ stiffness.

Girdlestone in 1947, followed by Taylor in 1951 were the first to describe tendon transfer procedures in the treatment LTD [36, 37]. Both authors transferred the FDL tendon to the extensors, inserting it dorsally at the level of the proximal phalanx. The main objective was to correct sagittal dorsiflexion contracture at the MTPJ by the tenodesis effect of the flexor-to-extensor transfer, as well as weakening the FDL pull at the tip of the toe. Other authors such as Parrish, Kuwada and Dockery later modified the original Girdlestone-Taylor technique in an attempt to optimize the direction of the transfer and increase its correction power [28, 38]. All of these techniques involve a relatively extensive dissection at the level of the proximal phalanx dorsally, to allow for the flexor-to-extensor tendon transfer. Even though multiple studies reported relatively high satisfaction rates with the procedures (60 to 90%), the rates of MTPJ stiffness (60%) and deformity recurrence (up to 16%) are substantial [36, 39] [40]. The flexor tenodesis procedure described here can be performed through a relatively small plantar approach with minimal tendon dissection, and an even smaller dorsal approach long enough to allow retraction of the extensor tendons and insertion of the implant. It also does not involve a flexor-to-extensor tendon transfer procedure, only a proximal tenodesis of the flexor tendons to the base of the proximal phalanx, what can be considered a less locally disturbing and more anatomical procedure. It is the author’s understanding that those differences could possibly explain the reasoning for less scar tissue formation and resultant less pronounced stiffness and relatively preserved MTPJ ROM observed with the flexor tenodesis technique.

Many authors have also relied on the combination of distal metatarsal shortening osteotomies and PIPJ resection arthroplasty/arthrodesis to correct rigid LTD and central metatarsalgia, with or without a flexor-to-extensor tendon transfer [41, 42]. Shortening the metatarsal and positioning the center of rotation of the metatarsal head slightly dorsal to the intrinsic musculature, it allows for a more anatomical vector with increased lever arm for the musculature to pull the proximal phalanx plantarly, correcting the MTPJ dorsiflexion deformity [43]. Fair to good functional results were reported, but a high percentage of complications have been associated with these procedures, particularly floating toes and MTPJ stiffness [18, 19, 44, 45]. Although biomechanically sound, the fact that the surgery is most commonly and traditionally performed open, and consists of an intraarticular osteotomy, it is inherently associated with increased scar tissue formation, dorsiflexion contracture (floating toes) and MTPJ stiffness [15]. The author’s preference in the setting of LTD and central metatarsalgia, with long 2nd, 3rd and 4th metatarsals, is to combine the flexor tenodesis technique with the percutaneous DMMO, an extra-articular and extra-capsular DMSO, potentially decreasing the incidence of floating toes and MTPJ stiffness. In the setting of a rigid contracture of the PIPJ, the authors would also recommend the use of PIPJ fusion technique, preferably avoiding the use of K-wire fixation to allow early weight bearing and MTPJ ROM exercises.

Several potential advantages with the use of the proposed technique and utilized implant can be highlighted. Relatively low invasive procedure with minimal surgical dissection and avoidance of a non-anatomical flexor to extensor tendon transfer; dynamic tenodesis effect of the flexor tendons in the base of the proximal phalanx, correcting the dorsiflexion deformity of the MTPJ; possibility of fine tuning the amount of tension applied in the flexor tendons, titrating the deformity correction; correction of flexible plantarflexion contraction of the interphalangeal joints by loosening the distal segment of the flexor tendons; possibility of resetting the tenodesis and fixation parts of the procedure if adequate/desired correction is not achieved; avoidance of K-wire fixation procedure and possible complications associated with it [31–33]; and apparently low incidence of floating toes and MTPJ stiffness, with maintained short-term deformity correction. However, no real conclusions can be drawn from our study, since only three patients were assessed during a short period of postoperative clinical follow-up. Additional data are needed to better define the success rate, safety and possible complications of the procedure, and must include the assessment of larger cohorts with longer postoperative follow-up. Prospective and comparatives studies with other used techniques are also needed.

In conclusion, in this surgical technical-tip study we described an alternative surgical technique to treat LTD, consisting of a flexor tenodesis procedure to the plantar aspect of the proximal phalanx, using a specific implant. The technique involves minimal surgical dissection, allows dynamic controlled correction of the sagittal plane MTPJ dorsiflexion deformity of the MTPJ, and can be performed in combination with other procedures such as DMSO and PIPJ fusions. Although adequate LTD correction and no complications were found in a case series of three patients, the lack of a larger sample, long-term evaluations and a comparative group limit the clinical applicability of the presented data. Additional long-term, prospective and comparative studies are needed.
