# Correction to: Flexor tenodesis procedure in the treatment of lesser toe deformities

**DOI:** 10.1007/s00402-021-04035-3

**Published:** 2021-07-14

**Authors:** Cesar de Cesar Netto, Eli L. Schmidt, Matthieu Lalevee, Nacime Salomao Barbachan Mansur

**Affiliations:** 1 Department of Orthopaedic and Rehabilitation, Carver College of Medicine, University of Iowa, 200 Hawkins Dr, John Pappan John Pavillion (JPP), Room 01066, Lower Level, Iowa City, IA 52242 USA; 2 Department of Orthopedics and Traumatology, Paulista School of Medicine, Federal University of Sao Paulo, Sao Paulo, SP Brazil

## Correction to: Archives of Orthopaedic and Trauma Surgery 10.1007/s00402-021-03942-9

The article Flexor tenodesis procedure in the treatment of lesser toe deformities, written by Cesar de Cesar Netto, Eli L. Schmidt, Matthieu Lalevee and Nacime Salomao Barbachan Mansur, was originally published electronically on the publisher’s internet portal on 11 May 2021 without open access. With the author(s)’ decision to opt for Open Choice the copyright of the article changed on 30 June 2021 to © The Author(s) 2021 and this article is licensed under a Creative Commons Attribution 4.0 International License, which permits use, sharing, adaptation, distribution and reproduction in any medium or format, as long as you give appropriate credit to the original author(s) and the source, provide a link to the Creative Commons licence, and indicate if changes were made. The images or other third party material in this article are included in the article’s Creative Commons licence, unless indicated otherwise in a credit line to the material. If material is not included in the article’s Creative Commons licence and your intended use is not permitted by statutory regulation or exceeds the permitted use, you will need to obtain permission directly from the copyright holder. To view a copy of this licence, visit http://creativecommons.org/licenses/by/4.0/.

The original article has been corrected.

